# Comparison of laparoscopic performance using low-cost laparoscopy simulators versus state-of-the-art simulators: a multi-center prospective, randomized crossover trial

**DOI:** 10.1007/s00464-025-11531-9

**Published:** 2025-01-30

**Authors:** Mark Enrik Geissler, Jean-Paul Bereuter, Rona Berit Geissler, Karl-Friedrich Kowalewski, Luisa Egen, Caelan Haney, Sofia Schmidt, Alexa Fries, Nathalie Buck, Juliane Weiß, Grit Krause-Jüttler, Jürgen Weitz, Marius Distler, Florian Oehme, Felix von Bechtolsheim

**Affiliations:** 1 Department of Visceral, Thoracic and Vascular Surgery, Faculty of Medicine, University Hospital Carl Gustav Carus, TUD Dresden University of Technology, Fetscherstraße 74, 01307 Dresden, Germany; 2 Department of Urology and Urosurgery, University Medical Centre Mannheim, University of Heidelberg, Mannheim, Germany; 3 Division Intelligent Systems and Robotics in Urology, German Cancer Research Center (DKFZ), Heidelberg, Germany; 4 DKFZ Hector Cancer Institute, University Medical Center Mannheim, Mannheim, Germany; 5 Centre for Tactile Internet with Human-in-the-Loop (CeTI), TUD Dresden University of Technology, Fetscherstraße 74, 01307 Dresden, Germany; 6 National Center for Tumor Diseases (NCT/UCC), Dresden, Germany; 7 German Cancer Research Center (DKFZ), Heidelberg, Germany; 8 Faculty of Medicine, University Hospital Carl Gustav Carus, Technische Universität Dresden, Dresden, Germany; 9 Helmholtz-Zentrum Dresden-Rossendorf (HZDR), Dresden, Germany

**Keywords:** Minimally invasive surgery, Laparoscopic simulators, Laparoscopic skill analysis, Simulation training, Surgical training, Randomized crossover trial

## Abstract

**Introduction:**

Simulator training is an efficient method for the development of basic laparoscopic skills. We aimed to investigate if low-cost simulators are comparable to more expensive box trainers regarding surgeons usability, likability, and performance.

**Methods:**

This multi-center, randomized crossover study included 16 medical students, seven abdominal surgeons, and seven urological surgeons. Participants performed four laparoscopic tasks (peg transfer, circle cutting, balloon resection, suture and knot) on both, a “Low cost trainer” (LCT) or a “high cost trainer” (HCT) in a randomized order. The primary endpoint was the subjective rating of both training simulators in terms of camera view, depth perception, movement of instruments, pricing, and usability for training. Secondary endpoints were force parameters, task completion time, surgical errors, and psychological workload.

**Results:**

Participants rated the LCT better concerning view (*p* < 0.001), depth perception (*p* = 0.003), pricing (*p* < 0.001), and usability for digital training (*p* < 0.001), but worse in terms of instrument movement (*p* = 0.004). Overall, the LCT was rated better than the HCT (*p* = 0.015). Regarding force parameters, participants showed a significantly lower force exertion on the HCT during the peg transfer task (*p* = 0.008). The force exertion in the other tasks were comparable between both trainers. Participants were significantly faster using the HCT during the peg transfer (*p* = 0.049) and significantly slower in balloon resection (*p* = 0.049) and suture and knot task (*p* = 0.026). The assessment of the participants’ workload showed no differences.

**Conclusion:**

The LCT was generally rated better than the HCT. The differences concerning force exertion and task completion time showed better results during peg transfer at the HCT but were generally inconclusive and without systemic advantage for either trainer. However, the LCT could be a promising and cost-effective augmentation for modern laparoscopic training.

**Graphical abstract:**

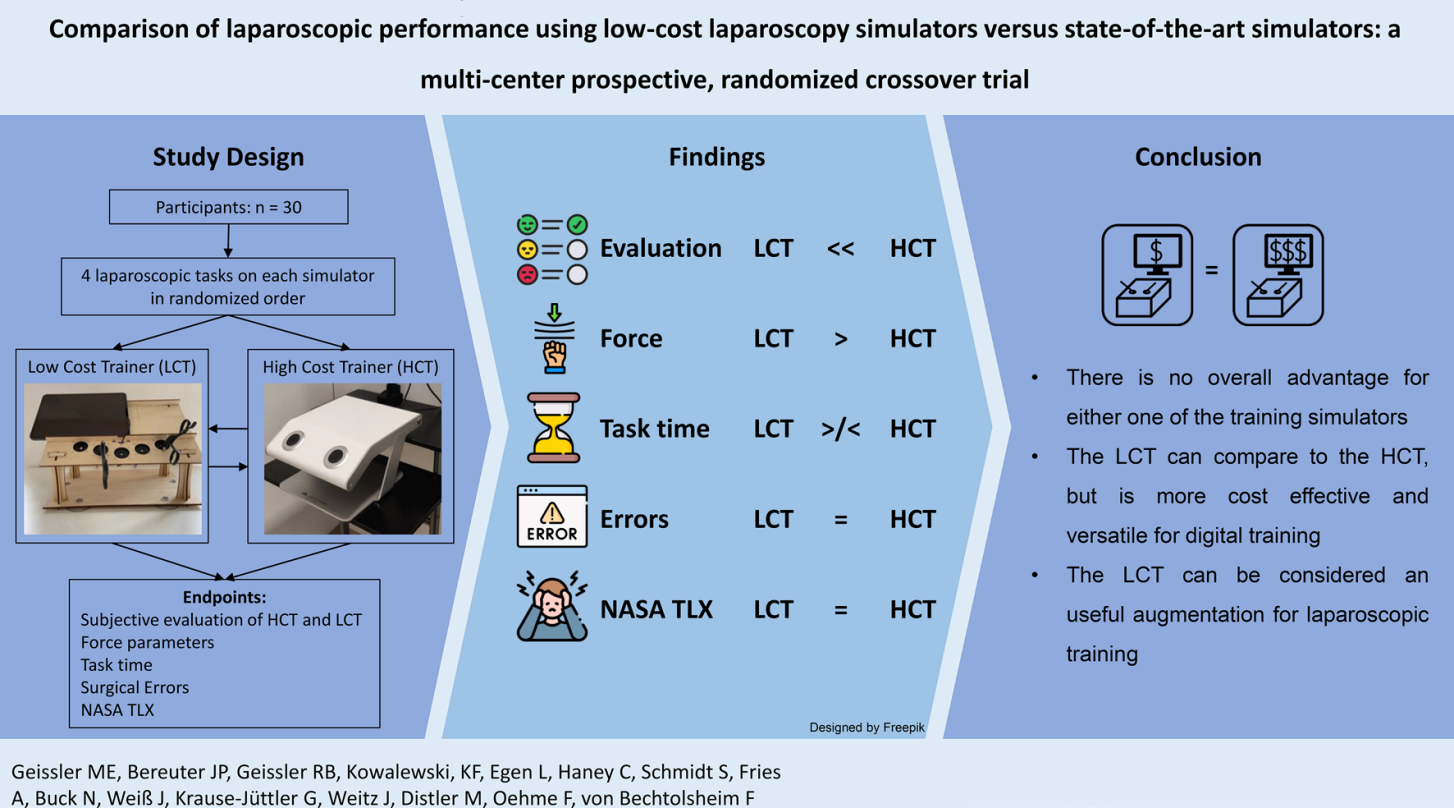

**Supplementary Information:**

The online version contains supplementary material available at 10.1007/s00464-025-11531-9.

Simulation is an efficient training tool for developing surgical novice’s basic laparoscopic skills [1, 2]. However, several factors are limiting the access to laparoscopic skill training [3]. For instance, simulator training takes place in simulation centers at hospitals and universities often occupying scarce room capacities. Adding to room and equipment capacity, is the personal cost to supervise training and observe room usage, increasing costs of structured training programs [4]. Additionally, most trainees have to use overtime for training purposes since it is mostly not included during normal working hours [3, 5]. Eventually current simulator systems and the respective equipment vary widely in price, ranging from hundreds to thousands of euros [6]. If laparoscopic and robotic virtual reality trainers (VR) are also taken into consideration prices are even higher [7]. These high costs and resource requirements limit the implementation and reduce accessibility to surgical training.

One way to overcome these barriers is to implement standardized training curriculums including the use of low-cost laparoscopy training simulators (LCT) [8, 9]. However, there are many different simulators currently available on the market [6]. One of the most prominent simulators are the FLS training simulator or the Lübecker Toolbox, both being rather high-cost training simulators (HCT). But also cheaper simulators such as the commercial "Laparoscopy Boxx," “iTrainer,” or self-made non-commercial simulator have been evaluated [6, 10–13]. Recent studies also indicated the possibility of low cost at-home training for laparoscopic surgery, opening up new personalized training possibilities [12, 14–17]. Such modalities support surgeons' initial acquisitions of basic laparoscopic skills as well as continuous development [14]. Additionally, it offers the possibility to decrease costs for already established training curricula [18]. At-home training also could facilitate skill retention, which might be important to cover extended periods without specialized surgical training (e.g., rotation to ICU, research time, parental leave) [16]. It may also overcome recently experienced challenges such as the Covid-19 pandemic and decreased exposure to operative procedures [15, 19]. Recently developed LCTs show great promise to democratize laparoscopic skill training [12]. The possibility of homemade simulators supports low-cost training even further. This allows trainees to choose their desired location and time of training [15]. In addition, low-cost simulators have the ability to decrease cost for surgeons and institutions [12]. Our study compares an available LCT with a HCT regarding the participants’ rating and performance using forceparameters, time, error, and workload parameters.

## Materials and methods

This randomized, crossover multi-center study was conducted in accordance with the Declaration of Helsinki [20]. The study was approved by the ethics committee of the Technische Universität Dresden (EK416092015), and participants gave written informed consent before participation.

### Participants

This study was performed at the University Hospital Carl Gustav Carus Dresden and the University Medical Centre Mannheim between April and August 2023. The study included medical students and physicians (general and urological surgeons). Before participation in the trial, all students participated in a modified *Fundamentals of Laparoscopic Surgery* (FLS) training curriculum [2]. Participating surgeons were not trained in a specific training curriculum. All participants were asked to perform four tasks in a standardized order (peg transfer, circle cutting, balloon resection, surgical suture and knot) twice, once using a LCT and once using a HCT. The order in which the simulators were used was randomized in order to rule out possible learning effects.

### Laparoscopic simulators and instruments:

To represent the LCT, the “Laparoscopy Boxx-Pro” (LCT) was chosen, which was developed by the Dutch company “Laparoscopy Boxx” (Outside the Boxx, Nijmegen, The Netherlands). The simulator consists of a wooden frame, can be stored and shipped easily and is simple to build up. The simulator alone costs 109€ and 309€ including instruments and does not have a built-in camera device and therefore requires a phone or tablet with a built-in camera (Supplementary Material 1). For this study a 9th generation iPad (Apple Inc., Cupertino, USA) was used as a camera and display. As HCT, the “Lübecker Toolbox'' (LTB Germany Ltd., Lübeck, Germany), a laparoscopic box trainer consisting mainly of metal and solid plastic material, was chosen (Supplementary Material 2). This simulator costs 3900€ not including instruments or a screen. For this study we used the standard camera connected to a screen. All exercises were performed using the same basic laparoscopic instruments such as forceps, laparoscopic scissors, and needle holders (Storz, Tuttlingen, Germany).

### Measurement

**Fig. 1:**
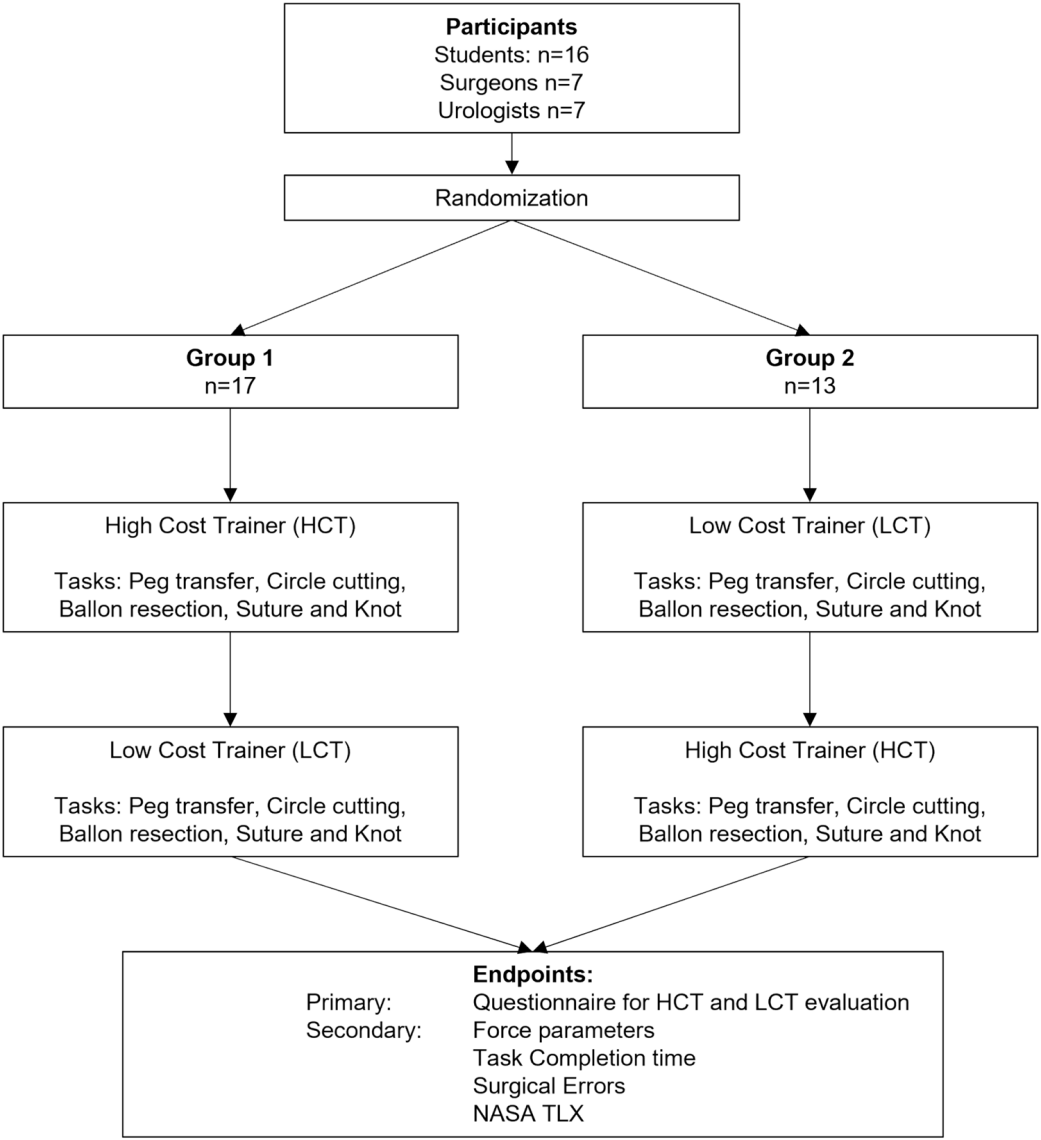
Flow chart of the study design

The test sessions were conducted in the laparoscopic training laboratory of the University Hospital Dresden and of the University Medical Centre Mannheim, which both ensured an environment that was absent of potentially biasing factors. For the measurement of laparoscopic force exertion, the ForceTrap device (Medishield B.V., Delft, The Netherlands) was utilized. Each task was attached on a platform linked to the ForceTrap device within the respective laparoscopic box trainer. The flow chart of the complete study design can be seen in Fig. 1.

### Primary endpoint: assessment of the simulators

For further evaluation of participants' perceived usability of the simulators, a comprehensive questionnaire was designed. This questionnaire requested aspects concerning the user experience such as camera view, depth perception, movement of instruments, pricing, and usability for digital training. Ratings were performed on a 5-point Likert scale with 1 being worst and 5 the best possible evaluation.

### Secondary endpoint

#### Force analyses

To analyze the applied force during the exercises we utilized the ForceTrap system (MediShield B.V., Delft, The Netherlands). In addition to the task needed for completion of the exercise, force-related parameters such as the peak force and the mean non-zero force were measured. The analyzed parameters were previously described in a study by Hardon and colleagues [21]:

- Mean non-zero force [N]: mean absolute force exerted by the instruments on the task platform during periods when force is not zero.
- Maximal force [N]: the highest absolute force exerted by the instruments on the task platform during the task.

#### Surgical errors

Surgical errors during the exercises were assessed and graded using the scheme by Bechtolsheim et al. [22] (Supplementary Material 3). peg transfer (triangle lost), circle cutting (cutting outside the line), and Balloon resection (Balloon perforation), indicating unsuccessful task completion. For the surgical knot task, three distinct error criteria were defined: imprecise stitching, incomplete suture approximation and faulty knot tightness. One individual blindly rated error occurrence for all tasks.

#### Task completion time

The time needed to complete each of the exercises was measured using the ForceTrap system (MediShield B.V., Delft, The Netherlands). The measurement started with the participants instrument movement and ended with its placement on a marked area in the operating field.

#### Psychological workload

To assess the participants’ psychological workload, the NASA Task Load Index (NASA-TLX) was used in this study [23]. This questionnaire is suitable to evaluate the perceived workload by utilizing a scoring system that consists of six different domains of workload (mental demand, physical demand, temporal demand, performance, effort, frustration) in relation to each other. For ranking the domains of interest, participants used a visual analog scale (VAS) to rate every domain on a scale (0–20). To quantify the perceived workload, the score of each domain was multiplied by an overall rating factor and cumulated into an overall score. The NASA-TLX was performed once for each conducted task during both the LCT and the HCT measurement.

### Statistical analysis

For the statistical analysis SPSS version 28 (IMB Corp, Armonk NY, USA) was utilized. First, continuous data was tested for normality by using the Kolmogorov–Smirnov test and by inspecting the frequency distributions. Concerning participant characteristics, values were summarized as mean values and standard deviations (SDs) for continuous variables or as absolute and relative frequencies for discrete variables. Depending on the data characteristics, the appropriate statistical test was utilized (paired student’s t-test, McNemar’s test, Wilcoxon rank test) to compare between both study groups. Missing data did not occur throughout the primary analysis. *p*-values < 0.05 were considered as statistically significant.

## Results

### Basic participant characteristics

In total, 30 participants were included in this multi-center, prospective randomized crossover non-inferiority trial. The participants' median age was 28.1 years (SD 5 years). Of these participants, fourteen were surgeons with experience in laparoscopy, comprising seven abdominal surgeons (23.3%) and seven urologists (23.3%), while the remaining sixteen participants (53.3%) were medical students. The study group demonstrated a balanced gender distribution, with seventeen male (56.7%) and thirteen female (43.3%) participants. Almost all participants were right-handed (96.7%). Most of the participants (70%) reported experience in laparoscopic surgery in the operating room (OR). Additionally, most of the students (59%) reported participation in basic surgical skills training, additionally to the performed laparoscopy course, during their college education (Table 1).

**Table 1:** Basic participant characteristics

Items	Values
Age, mean [years (SD)]	28.1 (4.97)
Profession [*n* (%)]	
Student	16 (53.3)
Abdominal surgeon	7 (23.3)
Urologist	7 (23.3)
Sex, number [*n* (%)]	
Male	17 (56.7)
Female	13 (43.3)
Dominant hand [*n* (%)]	
Right hand	29 (96.7)
Left hand	1 (3.3)
Surgical experience in college, number [*n* (%)]	18 (59)
Experience in MIS, number [*n* (%)]	21 (70)
Subjective level of proficiency, number [*n* (%)]	
Beginner	24 (80)
Intermediate	6 (20)
Proficient	0 (0)

### Primary endpoints

#### Assessment of the used simulators:

**Fig. 2:**
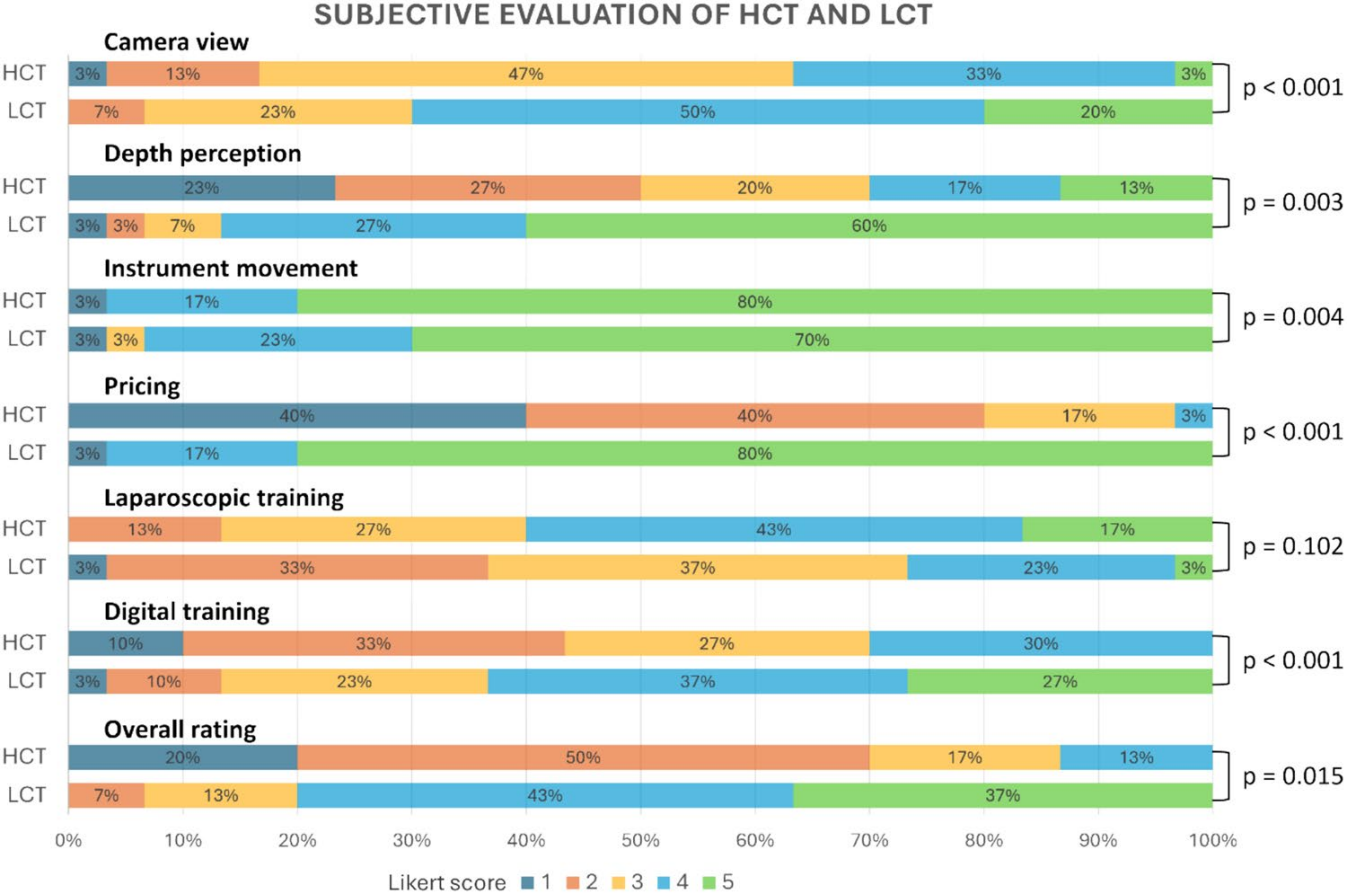
A stacked bar chart showing the subjective ratings based on a Likert scale (1 = worst to 5 = best evaluation) for both the LCT and HCT. Percentages in the bar are rounded

The assessment of the simulators showed several differences. The camera view of the LCT was rated significantly better compared to the HCT (LCT: 4.1 vs. HCT: 2.2; *p* < 0.001). In addition, the LCT was also rated significantly better regarding the depth perception (LCT: 3.7 vs. HCT: 2.8; *p* = 0.003), but worse concerning the movement of instruments (LCT: 2.9 vs. HCT: 3.6; *p* = 0.004). The pricing of the LCT was rated significantly better compared to the HCT (LCT: 4.7 vs. HCT: 1.8; *p* < 0.001). Concerning the usability of the simulators for laparoscopic training in general, no significant differences were observed in the participants' rating (LCT: 4.6 vs. HCT: 4.7; *p* = 0.102). Regarding the usability for digital laparoscopic training, the LCT was rated better than the HCT (LCT: 4.4 vs. HCT: 2.7; *p* < 0.001). In summary, the LCT was rated overall significantly better than the HCT (LCT: 3.8 vs. HCT: 3.2; *p* = 0.015) (Table 2; Fig. 2).

**Table 2:** Assessment of both simulators using a 5-point Likert scale (1 = worst; 5 = best evaluation)

items	LCT	HCT	*p*-value
	Mean (SD)	Mean (SD)	
Camera view	4.1 (0.89)	2.2 (0,94)	**< 0.001**
Depth perception	3.7 (1.08)	2.8 (1.00)	**0.003**
Movement of instruments	3.9 (0.92)	3.6 (0.93)	**0.004**
Pricing	4.7 (0.79)	1.8 (0.83)	**< 0.001**
Usability for laparoscopic training	4.6 (0.86)	4.7 (0.79)	0.102
Feasibility for digital training	4.4 (1.00)	2.7 (1.37)	**< 0.001**
Overall rating	3.8 (0.83)	3.2 (0.85)	**0.015**

Significant *p*-values are highlighted in bold

#### Subgroup analysis: simulator assessment

In subgroup analysis, students and physicians rated the LCT significantly better regarding the camera view (students: LCT: 4.2 vs. HCT: 1.9; *p* < 0.001; physicians: LCT: 3.9 vs. HCT: 2.6; *p* = 0.019), pricing (students: LCT: 3.7 vs. HCT: 1.7; *p* < 0.001; physicians: LCT: 4.6 vs. HCT: 1.9; *p* < 0.001), and feasibility for digital training (students: LCT: 4.5 vs. HCT: 3.2; *p* = 0.008; physicians: LCT: 4.2 vs. HCT: 2.3; *p* = 0.02). The depth perception was rated significantly better on the LCT by students (LCT: 4.1 vs. HCT: 2.5; *p* < 0.002) but not by physicians (LCT: 3.4 vs. HCT: 3.1; *p* = 0.5). The instrument movement on the other hand was rated significantly better on the HCT by students (LCT: 2.9 vs. HCT: 4.1; *p* < 0.009), whereas physicians’ rating showed no significant difference (LCT: 2.9 vs. HCT: 3.1; *p* = 0.27). There were no differences regarding the usability for laparoscopic training (students: LCT: 4.7 vs. HCT: 4.9; *p* = 0.32; physicians: LCT: 4.4 vs. HCT: 4.5; *p* = 0.16) and the overall rating (students: LCT: 4.0 vs. HCT: 3.2; *p* = 0.07; physicians: LCT: 3.6 vs. HCT: 3.1; *p* = 0.12) in either of the groups (Supplementary Material 4 and 5).

### Secondary endpoints

#### Maximal force analysis

The analysis of the applied peak force showed significant differences during the peg transfer task between the LCT and the HCT (LCT: 3.4 N vs. HCT: 2.9 N; *p* = 0.008). However, the peak force was not differing between the LCT and the HCT measurement for the circle cutting (LCT: 2.6 N vs. HCT: 2.8 N; *p* = 0.45), the balloon resection (LCT: 4.9 N vs. HCT: 5.9 N; *p* = 0.13), and the suture and knot task (LCT: 3.3 N vs. HCT: 3.5 N; *p* = 0.61) (Table 3).

**Table 3:** Comparison of task completion time and force exertion between the LCT and the HCT group

	LCT	HCT	*p*-value
	Mean (SD)	Mean (SD)	
Peg transfer			
Time [s]	192.15 (54.11)	175.3 (65.53)	**0.049**
Peak force [*N*]	3.4 (1.15)	2.85 (1.11)	**0.008**
Mean non-zero force [*N*]	0.88 (0.19)	0.73 (0.13)	**< 0.001**
Circle cutting			
Time [s]	210.44 (83.31)	232.37 (75.58)	0.192
Peak force [*N*]	2.64 (0.85)	2.82 (1.23)	0.453
Mean non-zero force [*N*]	0.76 (0.23)	0.77 (0.28)	0.805
Balloon resection			
Time [s]	198.59 (111.81)	223.08 (102.04)	**0.049**
Peak force [*N*]	4.85 (2.87)	5.94 (4.58)	0.131
Mean non-zero force [*N*]	1.14 (0.50)	1.22 (0.47)	0.484
Suture and knot			
Time [s]	253.64 (110.39)	293.95 (120.61)	**0.026**
Peak force [*N*]	3.28 (1.37)	3.47 (1.39)	0.607
Mean non-zero force [*N*]	0.83 (0.24)	0.84 (0.20)	0.805

Significant *p*-values are highlighted in bold

The peg task was performed with significantly lower force by students on the HCT (LCT: 3.7 N vs. HCT: 3.0 N; *p* = 0.03), whereas this was not observed in the physician. In contrast, physicians performed the cutting task with significantly less maximum force on the LCT (LCT: 2.3 N vs. HCT: 2.9 N; *p* = 0.01), which was not seen in the student group (Supplementary Material 3 and 4).

#### Mean non-zero force

Concerning the applied mean non-zero force the peg transfer task revealed significant differences between the LCT and the HCT measurement group (LCT: 0.9 N vs. HCT: 0.7 N; *p* < 0.001), while the circle cutting (LCT: 0.8 N vs. HCT: 0.8 N; *p* = 0.81), the balloon resection (LCT: 1.1 N vs. HCT: 1.2 N; *p* = 0.48) and the suture and knot task (LCT: 0.8 N vs. HCT: 0.8 N; *p* = 0.81) did not show differences for this parameter (Table 3).

The mean non-zero force was significantly lower on the peg task performed by students (LCT: 0.9 N vs. HCT: 0.7 N; *p* < 0.001) but not in the physician group. The other tasks showed no significant differences between within the subgroup analysis (Supplementary Material 6 and 7).

#### Task completion time

The peg transfer task was performed significantly faster using the HCT compared to the LCT (LCT: 192.2 s vs. HCT: 175.3 s; *p* = 0.049). In contrast, the task completion time measured for the balloon resection (LCT: 198.6 s vs. HCT: 223.1 s; *p* = 0.049) and suture and knot task (LCT: 253.6 s vs. HCT: 294.0 s; *p* = 0.026) was significantly shorter for the LCT compared to the HCT. The task completion time for the circle cutting task did not differ significantly between both simulators (LCT: 210.4 s vs. HCT: 232.4 s; *p* = 0.19) (Table 3).

Students performed the peg task faster on the HCT (LCT: 181.54 s vs. HCT: 155.12 s; *p* = 0.044), whereas physicians showed no significant difference in this task time. In contrast physician performed the ballon resection and surgical knot tasks significantly faster on the LCT (ballon resection: LCT: 196.88 s vs. HCT: 240.52 s; *p* = 0.026; surgical knot: LCT: 242.66 s vs. HCT: 308.19 s; *p* = 0.026), which could not be observed in students (Supplementary Material 6 and 7).

#### Surgical errors

The occurrence of surgical errors did not significantly differ between the LCT and the HCT in the peg transfer (LCT: 33% vs. HCT: 53.3%; *p* = 0.146), the circle cutting (LCT: 36.7% vs. HCT: 46.7%; *p* = 0.508), the balloon resection (LCT: 66.7% vs. HCT: 63.3%; *p* = 1.000), and the surgical suture and knot task (precision: LCT: 73.3% vs. HCT: 83.3%; *p* = 0.508; adaption: LCT: 3.3% vs. HCT: 10%; *p* = 0.625; tightness: LCT: 0% vs. HCT: 3.3%; *p* = 1.000) (Table 4).

**Table 4:** Comparison of error occurrence between the LCT and the HCT group

	LCT	HCT	*p*-value
Peg transfer**,** [*n*]	10	16	**0.031**
Circle cutting, [*n*]			0.25
> 5 mm	8	9	
> 10 mm	3	5	
Balloon resection, [*n*]			0.99
Micro-perforation	9	6	
Macro-perforation	11	13	
Suture and knot, [*n*]			0.25
Precise stitches	8	5	
Penrose adaption	16	16	
Knot tightness	6	9	

Significant *p*-values are highlighted in bold

With exception of significantly more errors by the physicians in the peg transfer task (LCT: 28.6% vs. HCT: 78.6%; *p* = 0.016), there were no further significant differences in error occurrence between simulators in either of the groups (Supplementary Material 8 and 9).

#### Psychological workload

Regarding the participants' perceived psychological workload, the overall NASA-TLX score was not differing between the LCT and the HCT measurement group for the peg transfer (LCT: 46.9 vs. HCT: 41.8; *p* = 0.1), the circle cutting (LCT: 47.5 vs. HCT: 46.5; *p* = 0.75), the balloon resection (LCT: 48 vs. HCT: 46.3; *p* = 0.59), and the suture and knot task (LCT: 53.2 vs. HCT: 52.6; *p* = 0.76) (Table 5).

**Table 5:** Comparison of NASA-TLX scores between the LCT and the HCT group

summary score	LCT	HCT	*p*-value
	Mean (SD)	Mean (SD)	
Peg transfer	46.89 (15.36)	41.8 (17.71)	0.104
Circle cutting	47.47 (16.28)	46.49 (17.03)	0.746
Balloon resection	48.07 (18.59)	46.27 (18.71)	0.586
Suture and knot	53.17 (20.32)	52.57 (20.59)	0.756

## Discussion

The increasing volume and complexity of minimally invasive surgical procedures requires well trained physicians. Mastering these skills is a continuous learning process and simulator training as a valuable means in established curricula [24]. Laparoscopic box trainers allow trainees to develop basic and advanced skills in a safe environment outside the OR [1, 2]. However, most laparoscopic simulator training requires a considerate financial investment while evidence concerning the evaluation of low-cost trainers is still limited [12, 25]. Therefore, the primary aim of this study was to evaluate the students’ assessment and acceptance of the LCT in comparison to the HCT. Secondly, the students’ laparoscopic performance was analyzed using both trainers to evaluate the usability of LCTs for digital training formats.

In this study, a low-cost laparoscopic box trainer was compared to a high-cost box trainer. In summary, and in line with Bökkerink et al. 2021, the subjective assessment of the simulators was in favor of the LCT [12]. For instance, participants rated the view and depth perception during the exercise significantly better using the LCT. This might be due to the fact that the LCT does not include a camera and use of a mobile phone or tablet camera is recommended. The additional autofocus and high-resolution display of new devices, such as the iPad used in this trial, allow a comfortable view and depth perception, adapting during the procedure. The HCT on the other hand has a built-in camera and is connected to a computer screen. This camera has a lower resolution and even with a larger screen cannot supplement the missing autofocus during the exercises.

Pricing is a crucial factor in the acquisition and implementation of simulators as a study conducted by Hertz et al. highlighted the importance of cost in the adoption of robotic training systems [26]. Our study similarly found that the pricing of simulators significantly favored the LCT over the HCT. At the time of the trial, the HCT with the basic exercise set cost 3,900€, while the LCT cost only 109€ with the basic exercise set. Although the LCT may require an additional camera system, the widespread availability of smartphones and tablets among students and young surgeons minimizes this cost. Therefore, the odds of successful implementation are very high.

Instruments are not included and need to be purchased additionally. However, this trial did not investigate the long-time usability and sustainability of both trainers, but it can be assumed that the sturdy HCT outlives the wooden LCT. This might especially be valuable in the clinical training facilities, which are frequently used by surgeons of different skill levels. This also might explain the significantly better ratings in terms of instrument movement in favor of the HCT, whereas the usability for training in general showed no significant differences between the two different simulators. This might be due to the larger size of the HCT, allowing for a larger operational field. However, the usability for digital training was rated significantly higher for the LCT. This could be attributed to the lower weight and transportability of the simulator. The wooden parts allow easy assembly and dismantling. The simulator can be transported in a book sized box.

Overall, the LCT was rated significantly better compared to the HCT. Especially the view and the feasibility for digital training was superior for the LCT. This might put LCTs in the spotlight of future digital simulator training approaches. Especially at-home training could support the implementation of laparoscopic simulation training in surgeon development. This training modality allows for increased personalization of the training by surgeons. Interestingly, the first digital training approaches were already shown by Joosten et al. [14, 16]. Joosten and colleagues showed that unsupervised at-home training can prevent the deterioration of laparoscopic skills [15].

Nevertheless, the subgroup analysis showed that physicians were less in favor of the LCT and only rated it better in pricing, feasibility for digital training and view. Furthermore, both groups, students and physicians, rated both simulators similarly concerning the overall rating and the usability for surgical training in general, underlining the usefulness of both trainers.

Our study did not show an overall significant impact of the used laparoscopic box trainer on the laparoscopic performance of surgical novices as well as surgeons. However, spotwise differences could be observed. For instance, the HCT was significantly better than the LCT in terms of task completion time and the applied peak and mean non-zero force for the peg transfer task. Interestingly, the LCT was superior to the HCT in the other three tasks regarding task completion time and also trends in favor for LCT could be observed concerning the applied peak and mean non-zero force. Nevertheless, the subgroup analysis revealed mixed results. Whereas students performed better at the HCT regarding the peg transfer, physicians showed better results on the LCT.

To explore potential effects of the used type of trainer, we employed a comprehensive set of endpoints. Despite the inclusion of quantitative evaluations, no systemic effects that would clearly favor one or the other device could be identified in our study. In contrast to a study conducted by Bökkerink and colleagues, there was no clear superiority of one simulator concerning laparoscopic performance [12].

Finally, we could show that both simulators are useful for laparoscopic training but have room for further improvement and evaluation of best use also for digital training formats.

## Strengths and limitations

Both the LCT and the HCT are constructed from different materials, exhibit varying levels of stability, and utilize distinct cameras and display technologies. These differences might raise concerns about their comparability. However, despite these variations, which are also reflected in their differing prices, both trainers demonstrate similarities in terms of force, time, error, and workload parameters.

To increase the comparability in this experimental setup, the ForceTrap system was used for measuring the task time as well as the applied mean and maximum force parameters.

The use of different cameras might explain the differences in participants’ assessment regarding the view during the exercises. This might limit the comparability of the view but represents the actual usage of these training devices. The stability and construction of the HCT were not further evaluated in this study. Especially regarding sustainability and product lifespan, this simulator might hold an advantage that was not studied further. Such aspects are also important to consider for the implementation of such simulators and need to be investigated separately.

Furthermore, we only analyzed two laparoscopic simulators. This limits the generalizability of the results for the overall class of low- and high-cost simulators. Nevertheless, in the particular case of our trial, low-cost laparoscopic simulators proved to be comparable. Therefore, further studies validating and comparing different simulators should be conducted.

Even though no general significant effect was found between both simulator types, the quality of these endpoints must be pointed out. The simplified representation of laparoscopic performance by reproducing the duration of individual tasks or analysis of major errors such as dropped peg does not reflect the complexity of laparoscopic surgery. Precisely, tissue handling is an important parameter that should be used as a relevant endpoint in performance analyses [27].

## Conclusion

Surgical simulator training should be accessible and effective in the means of producing sufficient learning curves, preparing surgical novices and improving experienced surgeons’ skills. The advantages of the LCT or HCT in some tasks were incoherent. Therefore, our study showed that inexpensive simulators are usually comparable to more expensive versions in terms of tissue handling. Regarding view, perception, and pricing, the low-cost simulator was rated even superior compared to the high-cost simulator. Nevertheless, the low-cost trainer showed some weakness in instrument movability, which should be improved in the future. Additionally, aspects such as sustainability and long-term usability should be evaluated further. The low-cost trainer seems a sufficient option for digital training. This could open new opportunities for continuous training and skill retention outside the hospital. Therefore, low-cost trainers might be feasible to extend the common training environment and allow for more personalized and flexible training at a low cost.

## Supplementary Information

Below is the link to the electronic supplementary material.

Supplementary file1 (DOCX 1991 KB)
