# Supplementary material for: Comparison of laparoscopic performance using low-cost laparoscopy simulators versus state-of-the-art simulators: a multi-center prospective, randomized crossover trial
Source: Surg Endosc. 2025 Jan 30;39(3):2016–25. doi: 10.1007/s00464-025-11531-9 (PMC11870972; doi:10.1007/s00464-025-11531-9)
Supplement: Supplementary file 1 — Supplementary file1 (DOCX 1991 KB) [file 464_2025_11531_MOESM1_ESM.docx]

**Supplementary Material:**

**Supplementary Material 1:** Display of the LCT: Laparoscopy Boxx” (Outside the Boxx, Nijmegen, The Netherlands) with an iPad (Apple Inc., Cupertino, USA) as camera and display.


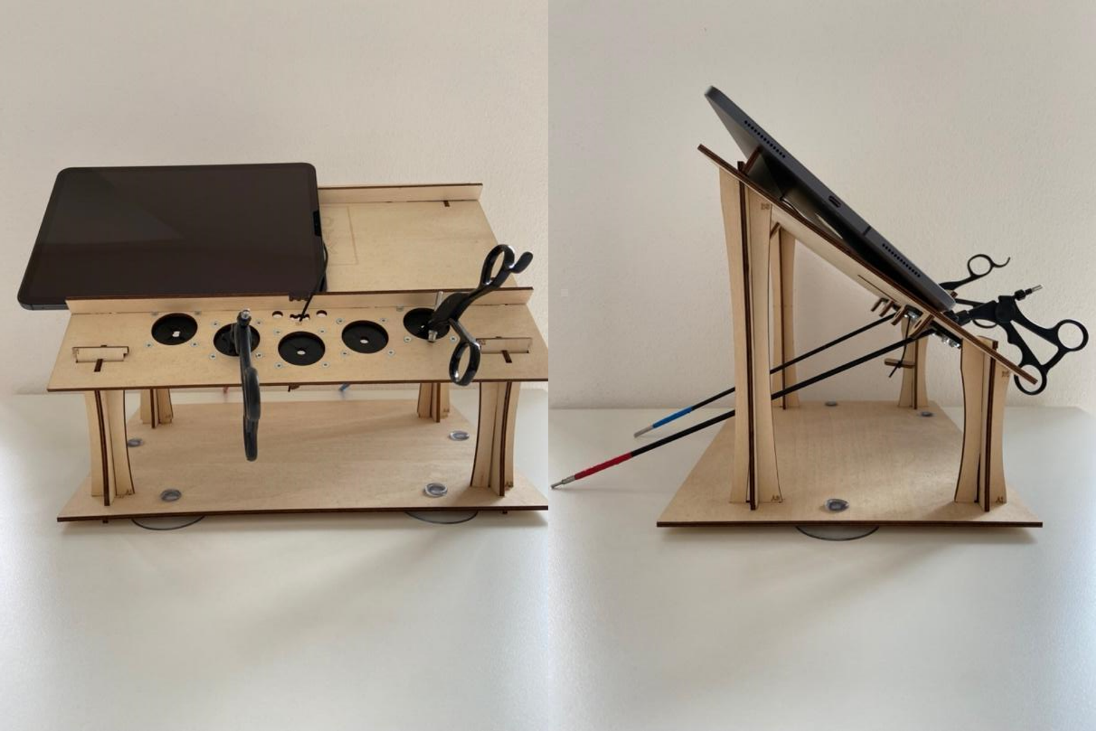


**Supplementary Material 2:** Display of the HCT: “Lübecker Toolbox'' (LTB Germany Ltd., Lübeck, Germany) with monitor


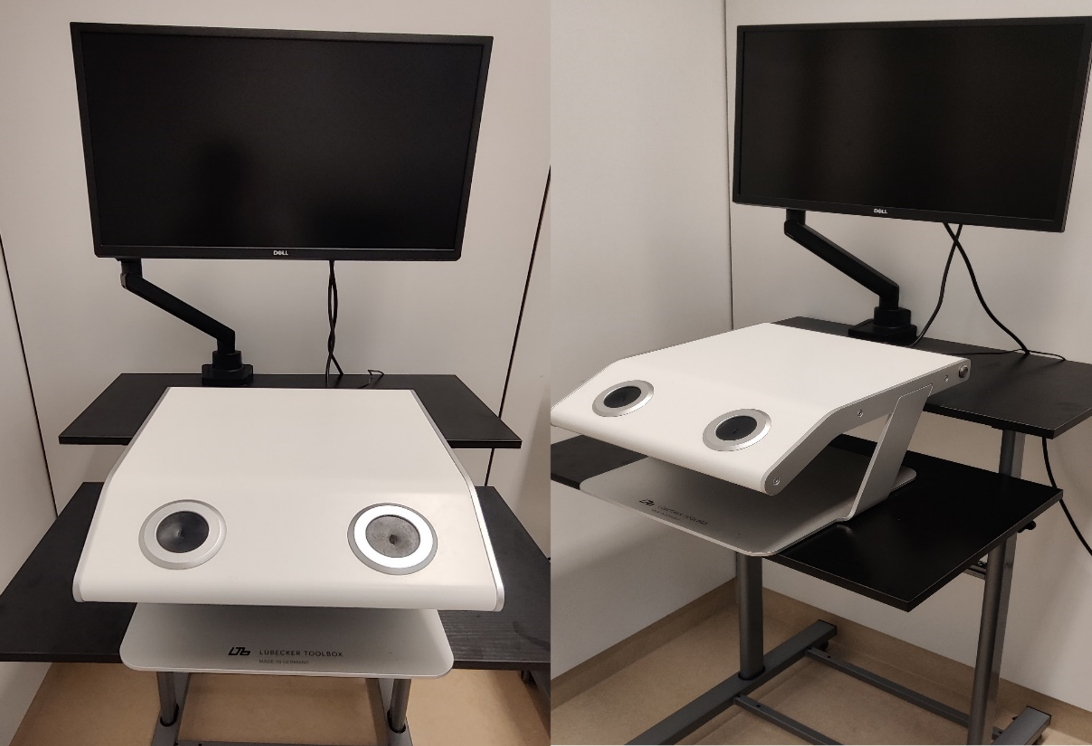


**Supplementary Material 3:** Error classification for each task. Significant errors were specifically defined for each task and recorded according to the following system which was described previously by Bechtolsheim et al. (Bechtolsheim et al., 2022).

| **error Definition** | **points** | **definition** |
| --- | --- | --- |
| Peg | 0 | no triangle dropped |
|  | 1 | per dropped triangle |
| Circle cutting | 0 | 0-5mm cut out of the margin |
|  | 1 | for each cut >5-10mm out of the margin |
|  | 2 | for each cut >10mm out of the margin |
| Balloon resection | 0 | no perforation |
|  | 1 | micro perforation- water leaks only under applied pressure |
|  | 2 | macro perforation- water leaks without applied pressure |
| Suture-precision | 0 | suture through both points on penrose |
|  | 1 | suture through one point on penrose |
|  | 2 | suture through no point on penrose |
| Suture-adaption | 0 | both sides of penrose touch |
|  | 1 | both sides of penrose adapt but do not touch |
|  | 2 | no adaption |
| Suture-tightness | 0 | knot tight under manipulation |
|  | 1 | knot visually tight but loosens under manipulation |
|  | 2 | knot visually loose |

**Supplementary Material 4:** Evaluation of HCT and LCT by students using a 5-point Likert scale (1 = worst; 5 = best evaluation).

| items | LCT  Mean (SD) | HCT  Mean (SD) | p-value |
| --- | --- | --- | --- |
| Camera view  Depth perception  Movement of instruments  Pricing  Use for training  Feasibility for digital training  Overall rating | 4.2 (0.68)  4.1 (0,85)  2.9 (1.00)  4.7 (0.45)  4.7 (0.45)  4.5 (0.73)  4.0 (0.82) | 1.9 (0,81)  2.5 (0,97)  4.1 (0.8)  1.7 (0.80)  4.9 (0.34)  3.1 (1.29)  3.2 (1.00) | **<0.001**  **0.002**  **0.009**  **<0.001**  0.317  **0.008**  0.065 |

**Supplementary Material 5:** Evaluation of HCT and LCT by physicians using a 5-point Likert scale (1 = worst; 5 = best evaluation).

| items | LCT  Mean (SD) | HCT  Mean (SD) | p-value |
| --- | --- | --- | --- |
| Camera view  Depth perception  Movement of instruments  Pricing  Use for training  Feasibility for digital training  Overall rating | 3.9 (1.07)  3.4 (1.08)  2.9 (0.86)  4.6 (1.08)  4.4 (1.15)  4.2 (1.25)  3.6 (0.84) | 2.6 (0,93)  3.1 (1.00)  3.1 (0.77)  1.9 (0.92)  4.5 (1.09)  2.3 (1.38)  3.1 (0.66) | **0.019**  0.523  0.271  **0.001**  0.157  **0.02**  0.118 |

**Supplementary Material 6:** Students subgroup analysis of task completion time and force exertion between the LCT and the HCT group. Significant p-values are highlighted in bold.

|  | LCT  Mean (SD) | HCT  Mean (SD) | p-value |
| --- | --- | --- | --- |
| Peg transfer  time [s]  peak force [N]  mean non-zero force [N]  Circle cutting  time [s]  peak force [N]  mean non-zero force [N]  Balloon resection  time [s]  peak force [N]  mean non-zero force [N]  Suture and knot  time [s]  peak force [N]  mean non-zero force [N] | 181.54 (42.63)  3.65 (1.05)  0.94 (0.15)  222.82 (74.99)  2.97 (0.916)  0.78 (0.275)  200.08 (130.69)  5.13 (2.876)  1.19 (0.41)  263.26 (109.08)  3.65 (1.56)  0.84 (0.219) | 155.12 (46.09)  3.00 (1.38)  0.73 (0.12)  237.21 (79.48)  2.77 (1.29)  0.75 (0.32)  207.82 (93.05)  6.07 (4.69)  1.20 (0.35)  281.48 (121.99)  3.46 (1.45)  0.84 (0.20) | **0.044**  **0.034**  **<0.001**  0.756  0.215  0.266  0.569  0.501  0.959  0.408  0.278  0.938 |

**Supplementary Material 7:** Physician subgroup analysis of task completion time and force exertion between the LCT and the HCT group. Significant p-values are highlighted in bold.

|  | LCT  Mean (SD) | HCT  Mean (SD) | p-value |
| --- | --- | --- | --- |
| Peg transfer  time [s]  peak force [N]  mean non-zero force [N]  Circle cutting  time [s]  peak force [N]  mean non-zero force [N]  Balloon resection  time [s]  peak force [N]  mean non-zero force [N]  Suture and knot  time [s]  peak force [N]  mean non-zero force [N] | 204.29 (64.31)  3.12 (1.22)  0.81 (0.22)  196.31 (92,69)  2.26 (0.59)  0.74 (0.18)  196.88 (90.48)  4.53 (2.93)  1.08 (0.59)  242.66 (114.95)  2.86 (1.03)  0.81 (0.27) | 198.37 (77.80)  2.68 (0.71)  0.71 (0.15)  226.84 (73.43)  2.88 (1.21)  0.79 (0,244)  240.52 (112.35)  5.79 (4.62)  1.23 (0.60)  308.19 (121.94)  3.49 (1.38)  0.83 (0.201) | 0.397  0.124  0.102  0.084  **0.011**  0.245  **0.026**  0.221  0.49  **0.026**  0.096  0.875 |

**Supplementary Material 8:** Students Subgroup analysis of error occurrence between the LCT and the HCT group. Significant p-values are highlighted in bold.

|  | LCT | HCT | p-value |
| --- | --- | --- | --- |
| Peg transfer [n] | 6 | 5 | 1.000 |
| Circle cutting [n] | 7 | 7 | 1.000 |
| Balloon resection [n] | 10 | 8 | .727 |
| Suture and knot [n]  precise stitches  penrose adaption  knot tightness | 10  0  0 | 15  1  0 | 0.63  1.000  n.a. |

**Supplementary Material 9:** Physician Subgroup analysis of error occurrence between the LCT and the HCT group. Significant p-values are highlighted in bold.

|  | LCT | HCT | p-value |
| --- | --- | --- | --- |
| Peg transfer [n] | 4 | 11 | **0.016** |
| Circle cutting [n] | 4 | 7 | 0.375 |
| Balloon resection [n] | 10 | 11 | 1.000 |
| Suture and knot [n]  precise stitches  penrose adaption  knot tightness | 12  1  0 | 10  2  1 | 0.625  1.000  1.000 |
